# Host Cell-Derived Extracellular Vesicles Regulate Iron Uptake in Recipient Macrophages during *Mycobacterium abscessus* Infection

**DOI:** 10.1021/acs.jproteome.5c00399

**Published:** 2025-10-22

**Authors:** Aidaly Daniela Ramos-Wolfley, Charlie A. Speelmon, Jing Zhang, Carlyn M. Guthrie, Olivia L. Clark, Steven D. Hartson, Lin Liu, Xuejuan Tan, Yong Cheng

**Affiliations:** † Department of Biochemistry and Molecular Biology, Oklahoma State University, Stillwater, Oklahoma 74078, United States; ‡ Oklahoma Center for Respiratory and Infectious Diseases, Oklahoma State University, Stillwater, Oklahoma 74078, United States; § Department of Physiological Sciences, Oklahoma State University, Stillwater, Oklahoma 74078, United States; ∥ Center for Genomics and Proteomics, Oklahoma State University, Stillwater, Oklahoma 74078, United States

**Keywords:** extracellular vesicles, macrophages, Mycobacterium abscessus, proteomics, intracellular iron

## Abstract

The role of host cell-derived extracellular vesicles (EVs) in host–pathogen interactions remains to be defined during mycobacterial infection. In this study, we characterized EVs from mouse RAW 264.7 cells uninfected or infected with *Mycobacterium abscessus* (*M*.*ab*), one of the most common nontuberculous mycobacterial (NTM) pathogens in humans. Our results show that *M*.*ab* infection increased the release of EVs but had no effect on the size distribution and total protein abundance of EVs from RAW 264.7 cells. Interestingly, EVs released by *M*.*ab*-infected RAW 264.7 cells significantly enhanced *M*.*ab* growth within recipient macrophages in cell culture. The proteomic analysis found that transferrin receptor was enriched in EVs from *M*.*ab*-infected RAW 264.7 cells compared to EVs from uninfected cells. Iron assay further indicates that EVs from *M*.*ab*-infected RAW 264.7 cells increased total iron level in recipient macrophages but not EVs from uninfected RAW 264.7 cells. However, iron abundance within EVs remained similar across both conditions. Finally, EVs from *M*.*ab*-infected RAW 264.7 cells failed to improve *M*.*ab* growth within recipient macrophages in the presence of an iron-chelating agent, deferoxamine, in cell culture. Therefore, our data suggest that *M*.*ab*-infected mouse macrophages likely regulate *M*.*ab* growth by upregulating iron uptake in recipient cells via released EVs.

## Graphical abstract

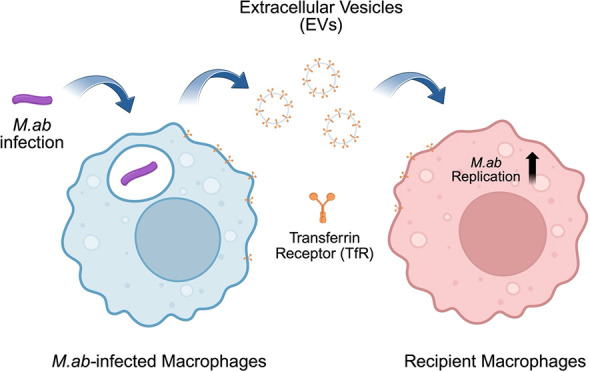

## Introduction

*Mycobacterium abscessus* (*M*.*ab*) is a member of the nontuberculous mycobacteria (NTM), which includes a diverse set of environmental bacteria typically found in soil, dust, water, and other natural sources. While *M*.*ab* is generally harmless to healthy individuals, under certain conditions, *M*.*ab* can cause severe respiratory infections, particularly in patients with underlying pulmonary diseases such as chronic obstructive pulmonary disease (COPD) and cystic fibrosis (CF).^1–5^ Similar to *Mycobacterium tuberculosis* (*M*.*tb*), the causative agent of tuberculosis (TB) in humans,^6,7^ the pathogenesis of *M*.*ab* is complex, as it has developed several mechanisms to survive and proliferate within host cells, especially in alveolar macrophages, which are the primary immune cells responsible for defending the lungs against pathogens.^8^ Macrophages are professional phagocytes, which actively engulf and destroy invading microorganisms by utilizing various antimicrobial mechanisms, including antimicrobial peptides, reactive oxygen species (ROS), reactive nitrogen species (RNS), phagosome-lysosome fusion and autophagy.^9^ However, *M*.*ab* has evolved several strategies to resist these defenses, including the ability to inhibit phagosome-lysosome fusion in macrophages.^8^ The interaction between *M*.*ab* and macrophages not only allows the bacteria to persist within the host cells but also contributes to chronic infection and inflammation, leading to the progression of *M*.*ab*-associated pulmonary diseases. Treatment of *M*.*ab* pulmonary infection is challenging in humans due to the lack of effective drug regimens and the prolonged treatment duration (18–24 months).^10,11^ Therefore, understanding the molecular interactions between *M*.*ab* and macrophages is critical for developing effective therapies and improving clinical outcomes for patients with chronic *M*.*ab* pulmonary infection.

Cell-to-cell communication plays a crucial role in the host’s response to bacterial infection, helping to coordinate immune responses, tissue repair, and inflammation. Cell-to-cell communication is regulated through multiple mechanisms, such as the secretion of cytokines, which act as signaling molecules that facilitate communication between immune cells,^12^ and the release of extracellular vesicles (EVs), which serve as vehicles for transporting biomolecules across cells.^13^ EVs are a group of membrane-bound vesicles with a lipid bilayer, that mediate cell-to-cell communication and regulate various biological processes, such as antigen presentation and pathogen clearance in immune cells.^13,14^ Mammalian cells release different types of EVs, such as exosomes and microvesicles (MVs) based on their biogenesis pathways.^13,14^ Exosomes are EVs originating from the multivesicular body (MVB) that are formed via the inward budding of the endosomal membrane, and released to extracellular environment via the fusion of MVB with the cytoplasmic membrane. MVs are generated through direct outward budding of the cytoplasmic membrane. EVs carry proteins, lipids, RNA, and other molecules derived from the parent cells, and can be taken up by recipient cells to modulate their function.^13,15^ In the context of bacterial infection, these vesicles may serve as carriers of pathogen-associated molecular patterns (PAMPs), such as bacterial proteins, RNAs and lipids, and regulate host immunity via activating recipient immune cells or uninfected host cells.^13,16,17^ Macrophage-released EVs have been widely studied previously in host response to mycobacterial infection, especially in the cellular response to *M*.*tb* infection.^13,18^ In contrast to *M*.*tb* infection, limited studies have focused on macrophage-derived EVs in the context of mycobacterial infection caused by NTM pathogens, such as *M*.*ab*.

In this study, we isolated EVs from either uninfected or *M*.*ab*-infected mouse macrophages cultured *in vitro* and characterized their proteomic profiles. We also investigated how the release of EVs during mycobacterial infection might alter cellular interactions and influence the host’s response to *M*.*ab* infection. Our results reveal that EVs released by *M*.*ab*-infected macrophages significantly enhance the intracellular growth of *M*.*ab* within recipient macrophages in cell culture. This effect is associated with an EV-mediated upregulation of intracellular iron levels in recipient macrophages, which are critical for bacterial survival and replication within host cells.^19,20^ These findings suggest that *M*.*ab*-infected macrophages actively release EVs that not only reflect the changes in host cell metabolism but also contribute to bacterial pathogenesis by altering key host cellular processes such as iron homeostasis.

## Materials and Methods

### Mammalian Cell Culture

RAW 264.7 cells were cultured at 37 °C and 5% CO_2_ in Dulbecco’s Modified Eagle Medium (DMEM) (Cat. No. SH30243.01; HyClone) supplemented with 10% (v/v) fetal bovine serum and streptomycin and penicillin (Cat. No. SV30010; HyClone) at a final concentration of 100 U/mL as we did previously.^21^

### Bacterial Culture

*M*.*ab* ATCC 19977 was grown in Middlebrook 7H9 broth (Cat. No. M198; HiMedia Laboratories) supplemented with 10% (v/v) OADC (oleic acid-albumin-dextrose-catalase, 0.05% Tween 80) at 37 °C.

### EVs Isolation and NanoSight Analysis

RAW 264.7 cell line was either left uninfected or infected with *M*.*ab* ATCC 19977 at a multiplicity of infection (MOI) of 5 for 4 h in complete DMEM medium. After the infection, the cells were washed three times with prewarmed 1× PBS to remove any extracellular bacteria. Subsequently, the cells were incubated in EVs-free DMEM medium for 72 h at 37 °C and 5% CO_2_ to allow for the collection of cell-secreted EVs as described in our previous studies.^21,23^ The EV-free DMEM medium was prepared as we did previously.^21,22^ Host cell-released EVs were harvested from the cell culture supernatant via a differential centrifugation procedure as described previously.^23^ Cell debris and apoptotic bodies were sequentially removed at 1000*g* and 4 °C for 15 min, and 2000*g* and 4 °C for 20 min. The EVs were finally isolated at 10,000*g* and 4 °C for 30 min, followed by three washes with precold 1× PBS. Isolated EVs were analyzed by the NanoSight NS300 (Malvern Panalytical, UK) to determine the yield of EV isolation. The protein abundance was determined using Pierce BCA Protein Assay Kits (Cat. No. 23227; Thermo Scientific). For some experiments, isolated EVs were further purified using sucrose gradient centrifugation as described previously.^24^

### Transmission Electron Microscopy (TEM)

EV samples were analyzed by TEM as we did previously.^1^ Briefly, the isolated EVs were applied to carbon-film TEM grids (Electron Microscopy Sciences, Hatfield, PA, USA), and then stained with 2.5% uranyl acetate. The stained samples were subsequently imaged using transmission electron microscope (JEOL Ltd., Akishima, Tokyo, Japan) at the Oklahoma State University Microscopy Core Facility.

### Mycobacterial Survival Assay in Macrophages

RAW 264.7 cells were either left untreated or pretreated with purified EVs (EVs/Cell = 100:1) with/without iron chelator, deferoxamine (Sigma-Aldrich, Cat. No. D9533; 100 μM), for 24 h *in vitro* at 37 °C and 5% CO_2_. After pretreatment, the cells were infected with *M*.*ab* (MOI = 5) for 1 h at 37 °C and 5% CO_2_. Following the infection, cells were washed three times with DMEM to remove any remaining extracellular *M*.*ab*. The cells were then incubated for additional periods (1 and 72 h) at 37 °C and 5% CO_2_. After incubation, the cells were washed three times with 1× PBS and subsequently lysed with 0.05% SDS. Cell lysates were serially diluted in 1× PBS and plated on Middlebrook 7H10 agar plates (Cat. No. 61000–050; HiMedia) supplemented with 10% (v/v) OADC. *M*.*ab* colonies were counted after 3–5 days of incubation at 37 °C.

### Quantitative RT-PCR

RAW 264.7 cells were treated with EVs isolated from uninfected or *M*.*ab*-infected macrophages (EVs/Cell = 100:1) for 24 h *in vitro* at 37 °C and 5% CO_2_. The cellular total RNA was isolated using Monarch’s Total RNA Miniprep Kit (Cat. No. T20105; New England Biolabs) according to the manufacturer’s instruction. The cDNA was synthesized using AMV reverse transcriptase (Cat. No. B0277A; New England Biolabs) as we did previously.^25^ Quantitative PCR was then performed on the Roche LightCycler 480 real-time PCR system (Qiagen) using luna universal master mix (Cat. No. M3003E; New England Biolabs) and primers for *Tfrc* (FW: 5′-TATTGTAAGCGTGTAGAACAAAAA-3′; RE: 5′-GTTTTGAGGTCTGCCC AATATA-3′), and GAPDH (FW: 5′-TCGTCCCGTAGACAAAATGG-3′; RE: 5′-TTGAGGTCAATG AAGGGGTC-3′).

### Proteomic Analysis for EVs

Purified EVs were analyzed using a “high/low”-mass accuracy data-dependent HCD MS/MS in a quadrupole-Orbitrap mass spectrometer (Fusion model, Ther- mofischer) in the proteomics core facility at Oklahoma State University as we did previously.^19,21^ Raw data were mapped to a reference database containing 55,334 *Mus musculus* proteins (UniProt database UP000000589) using Perseus (version 2.0.10.0). Similar to our previous study,^21^ the following criteria were applied in MaxQuant: (i) Fixed modification: Carbamidomethyl of cysteine (Cys), and (ii) Variable modifications: oxidation of methionine (Met), acetylation of protein N-termini, and chemical cyclization of glutamine (Gln) residues to pyroglutamate if they are at the n-terminus of the peptide. For the PCA analysis, the LFQ intensities of the identified proteins were log_2_ transformed, and normalized using the package “prcomp” in R. The first two principal components, accounting for the largest proportion of variance, were visualized using scatterplots generated in R with ggplot2 (v3.5.1).^26^ To identify differentially regulated proteins, the proteins exhibiting a fold change >1.5 and *p* < 0.05 were considered to be differentially enriched. To further interpret the biological significance of these differentially regulated proteins, a protein pathway analysis was conducted using Metascape custom analysis for *M. musculus*, focusing on Gene Ontology (GO) Biological Processes, KEGG Pathways and GO Cellular Components, as described in our previous work.^19,21,27^

### Western Blot Analysis

The assay was performed as we did previously.^28^ EVs from uninfected or *M*.*ab*-infected RAW 264.7 cells were run on an SDS-PAGE gel and transferred to a PVDF membrane. The membranes were incubated with rabbit anti-CD71 (Abcam, Cat. No. ab214039) or mouse anti-β-actin antibody (Biolegend, Cat. No. 643802), followed by goat anti-rabbit IgG (Invitrogen, Cat. No. 31460) or anti-mouse IgG secondary antibody (Invitrogen, Cat. No. G21040).

### Intracellular Iron Measurement

RAW 264.7 cells were untreated or treated with EVs isolated from uninfected or *M*.*ab*-infected macrophages (EVs/Cell = 100:1) for 24 h *in vitro* at 37 °C and 5% CO_2_. The intracellular iron abundance in macrophages was determined using the iron assay kit (Sigma-Aldrich, Cat. No. MAK025) following the manufacturer’s instruction as we did previously.^19^

### Statistical Analysis

Data were analyzed using PRISM GraphPad (Version 9.5.0) via Student’s paired *t* tests or one-way ANOVA with Tukey’s multiple comparisons test; *p* ≤ 0.05 was considered significant.

### Data Availability

The mass spectrometry proteomics data have been deposited to the ProteomeXchange Consortium via the PRIDE^40^ partner repository with the data set identifier PXD062573.

## Results

### *M*.*ab* Infection Increases EVs Release by Mouse RAW 264.7 Cells in Cell Culture

To determine the effect of *M*.*ab* infection on EV biogenesis in macrophages, we isolated EVs from either uninfected or *M*.*ab*-infected RAW 264.7 cells in cell culture. As shown in Figure 1A, Nanosight analysis indicates that *M*.*ab* infection had no effect of EV size distribution. A similar result was observed in the TEM analysis for purified EVs from uninfected or *M*.*ab*-infected RAW 264.7 cells (Figure 1B). However, *M*.*ab* did increase the release of EVs by RAW 264.7 cells. As seen in Figure 1C, EV release was increased by about 50% in *M*.*ab*-infected RAW 264.7 cells when compared to uninfected RAW 264.7 cells. In contrast, a similar level of the total protein abundance was detected in EVs isolated from uninfected or *M*.*ab*-infected RAW 264.7 cells (Figure 1D). We also purified EVs using sucrose gradient centrifugation as described previously.^24^ As shown in Figure 1E, a similar abundance of total proteins was detected in EVs from either uninfected or *M*.*ab*-infected RAW 264.7 cells.

**Figure 1:**
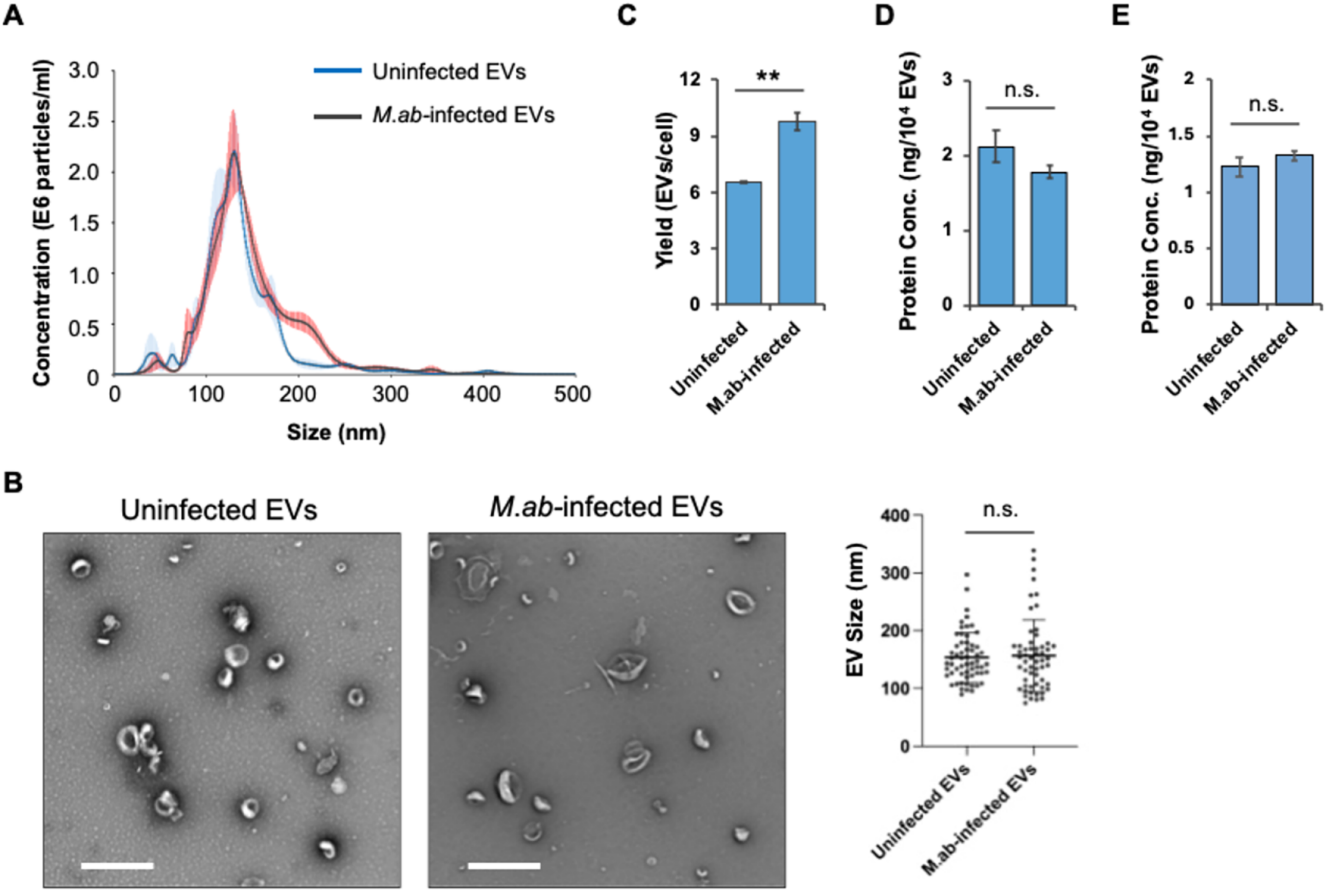
Characterization of purified EVs. (A) NanoSight analysis for EVs isolated from uninfected (Uninfected EVs) or *M*.*ab-*infected (*M*.*ab*-infected EVs) RAW 264.7 cells. Solid line indicates average number of particles (*n* = 5 technique repeats). Red and blue shading demonstrates standard deviation for each sample. (B) Representative image of transmission electron microscopy analysis for purified EVs (*n* = 5 random fields/sample) and related quantification based on multiple EVs under random fields (*n* = 60 EVs/sample). Scale bar: 500 nm. (C) EVs yield from uninfected or *M*.*ab-*infected RAW 264.7 cell culture. Total EVs yield was determined by NanoSight NS300 and calculated based on the cell density used for vesicle isolation. (D) Protein quantification by BCA analysis for EVs isolated from uninfected or *M*.*ab*-infected RAW 264.7 cells using differential centrifugation method. (E) Protein quantification by BCA analysis for EVs that were further purified using sucrose gradient centrifugation. Data are representative of three independent experiments. The results in (C–E) are mean ± SD (*n* = 3 biological repeats/group). n.s., not significant; **p < 0.01 by two-tailed Student’s *t* test.

### Proteomic Analysis for Isolated EVs from Uninfected or *M*.*ab*-Infected RAW 264.7 Cells

To better understand the role of macrophage-released EVs on the host–pathogen interaction in the context of mycobacterial infection, we first performed the proteomic analysis for EVs that were isolated from uninfected or *M*.*ab*-infected RAW 264.7 cells in cell culture. As shown in Figure 2A, principal component analysis (PCA) reveals that the proteomic profiles of the samples under each condition (*M*. *ab*-infected vs uninfected EVs) are similar. We further identified 5 unique host proteins (Table 1) in EVs isolated from *M*.*ab*-infected RAW 264.7 cells, and 124 unique host proteins (Table S1) in EVs from uninfected RAW 264.7 cells (Figure 2B). 839 overlapping host proteins were detected in EVs from uninfected or *M*.*ab*-infected RAW 264.7 cells (Figure 2B). Among those overlapping host proteins, the abundance of 58 host proteins (Table 2) was elevated in EVs isolated from *M*.*ab*-infected RAW 264.7 cells when compared to EVs from uninfected RAW 264.7 cells. In contrast, the abundance of 73 host proteins (Table S1) was downregulated in EVs isolated from *M*.*ab*-infected RAW 264.7 cells compared to EVs from uninfected RAW 264.7 cells (Figure 2C). The abundance of 708 detected host proteins is comparable in EVs from uninfected and *M*.*ab*-infected RAW 264.7 cells (Figure 2C and Table S1). The volcano plot in Figure 2D shows the differentially regulated proteins that were included in Figure 2C. As expected, we detected a list of host proteins that are generally identified in all types of EVs^14^ (Figure S1 and Table S2). We also identified a group of mycobacterial proteins in EVs released by *M*.*ab*-infected RAW 264.7 cells but not EVs from uninfected Cells (Table S3).

**Figure 2:**
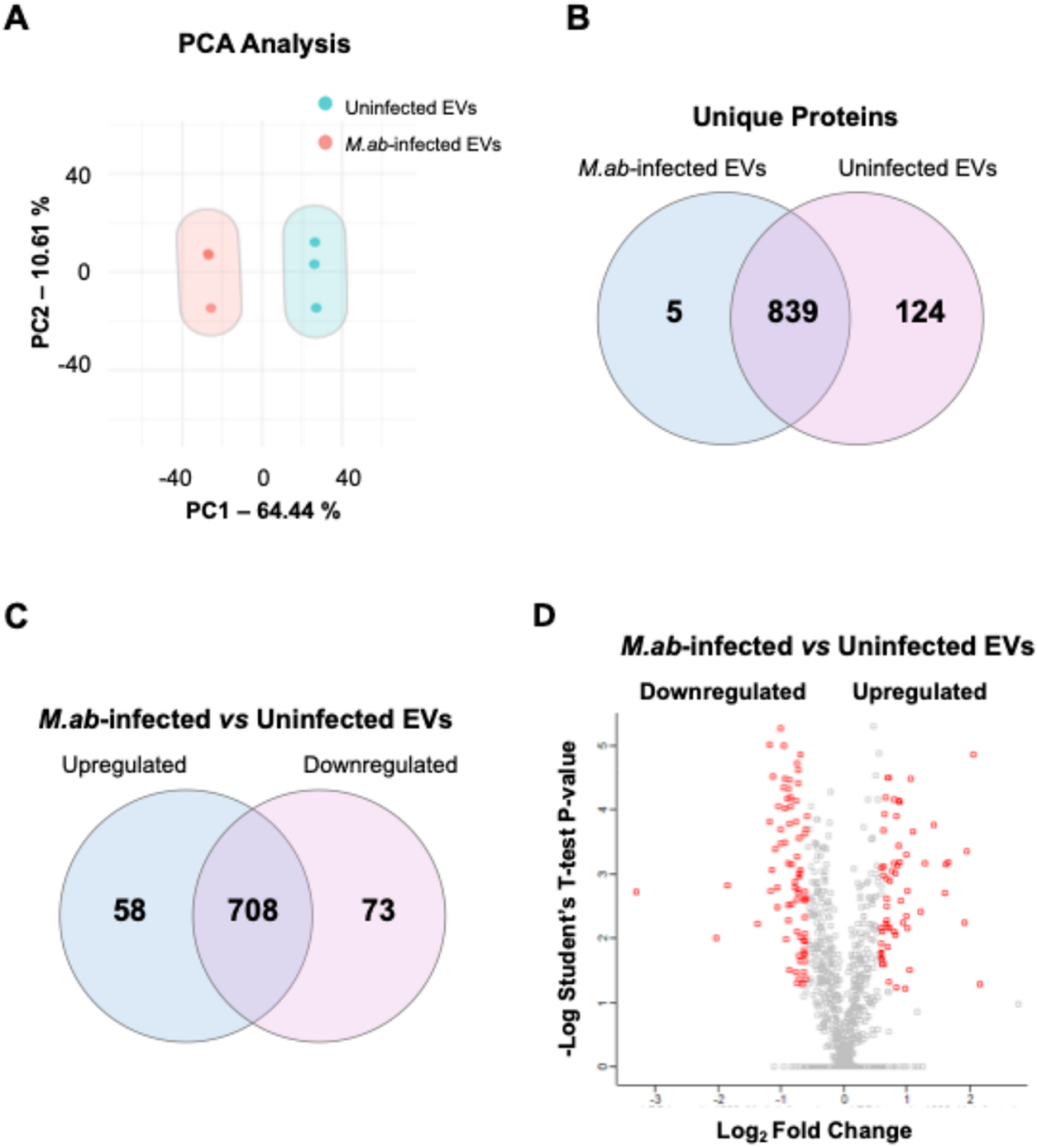
Proteomic analysis for purified EVs. (A) PCA analysis for the proteomic data. (B) Venn diagram for host proteins identified in EVs isolated from uninfected or *M*.*ab*-infected RAW 264.7 cells. (C) Venn diagram for overlapping host proteins that are differentially enriched in EVs isolated from uninfected and *M*.*ab*-infected RAW 264.7 cells (Cutoff: Fold Change >1.5 and *p* < 0.05). (D) Volcano plot for host proteins differentially enriched in EVs isolated from uninfected or *M*.*ab*-infected RAW 264.7 cells.

**Table 1:** Host Proteins Unique in *M*.*ab*-Infected EVs vs Uninfected EVs

protein names	gene names	majority protein IDs
eukaryotic translation initiation factor 3 subunit G	Eif3g	Q9Z1D1
60S ribosomal protein L9	Rpl9	A0A0G2JES3; A0A140T8T4; P51410; A0A0G2JFQ3; D3YZT0; D3Z629
monocarboxylate transporter 4	Slc16a3	P57787
peptidyl-prolyl cis–trans isomerase FKBP1A	Fkbp1a	P26883
Titin	Ttn	E9Q8N1; E9Q8K5; A0A5K1VVQ9; A2ASS6; F7CR78; A0A5K1VVQ1

**Table 2:** Mouse Proteins Highly Enriched in EVs from *M*.*ab*-Infected Macrophages

protein names	gene names	log_2_ fold change	–log *p*-value	majority protein IDs
osteopontin	Spp1	2.04400062561035	4.86182826578053	F8WIP8; P10923
amino acid transporter; neutral amino acid transporter B(0)	Slc1a5	1.95211601257324	3.36367221600132	Q9ESU7; P51912
interferon-induced transmembrane protein 3	Ifitm3	1.91171264648438	2.24545813289086	Q9CQW9
sequestosome-1	Sqstm1	1.64423179626465	3.1769883000148	Q64337; D3YZJ1
syndecan-4	Sdc4	1.60978317260742	3.15882511676953	O35988
sodium-coupled neutral amino acid transporter 2	Slc38a2	1.60745811462402	2.69870716167868	Q8CFE6
transferrin receptor protein 1	Tfrc	1.41882705688477	3.76643410905307	Q62351; Q8C872
protein CREG1	Creg1	1.29401397705078	3.17269119565696	O88668; K4DI63; J3QP41
translationally controlled tumor protein	Tpt1	1.2086181640625	2.41398741718999	P63028; D3YU75
CD166 antigen	Alcam	1.09227561950684	3.67095927253926	E9Q4G8; E9Q3Q6; Q61490
4F2 cell-surface antigen heavy chain	Slc3a2	1.0536994934082	4.48157739560875	P10852; A0A0U1RP98
monocarboxylate transporter 1	Slc16a1	1.03939628601074	1.51103639520649	P53986
sodium/potassium-transporting ATPase subunit β-3	Atp1b3	1.00730323791504	2.74039954586684	P97370
ragulator complex protein LAMTOR5	Lamtor5	1.00613212585449	2.15233908237208	Q9D1L9; G3UW70
barrier-to-autointegration factor; barrier-to-autointegration factor, N-terminally processed	Banf1	0.994245529174805	2.34294229387194	O54962
cathepsin B; cathepsin B light chain; cathepsin B heavy chain	Ctsb	0.993902206420898	3.30368678059513	P10605
Rho GDP-dissociation inhibitor 2	Arhgdib	0.966434478759766	1.22870874764035	Q61599
solute carrier family 2, facilitated glucose transporter member 1	Slc2a1	0.941097259521484	2.24503649281436	P17809
large neutral amino acids transporter small subunit 1	Slc7a5	0.90849494934082	2.58856571327426	Q9Z127
galectin-3-binding protein	Lgals3bp	0.891365051269531	3.18715817835325	Q07797
CD44 antigen	Cd44	0.885757446289063	4.12728429223604	Q3U8S1; A2APM5; A2APM3; A2APM4; E9QKM8; Q80X37; A2APM1; A2APM2; P15379
plexin-B2	Plxnb2	0.870326995849609	3.44243533488941	B2RXS4
ragulator complex protein LAMTOR1	Lamtor1	0.862848281860352	4.13593216723782	Q9CQ22; A0A0A6YX02
high affinity immunoglobulin epsilon receptor subunit γ	Fcer1g	0.854934692382813	3.14119873117423	P20491
cyclin-dependent kinase 1	Cdk1	0.838760375976563	1.23966001623262	D3Z2T9; P11440
tyrosine-protein phosphatase nonreceptor type substrate 1	Sirpa	0.834499359130859	3.90919996450559	P97797
integrin α-M	Itgam	0.821626663208008	3.010915191065	E9Q604; G5E8F1; E9Q5K8; Q3U1U4; A0A0R4J1B4; P05555
protein S100-A10	S100a10	0.814363479614258	2.06035900070648	P08207
CD180 antigen	Cd180	0.80729866027832	2.10531902779156	Q62192
tyrosine-protein kinase Lyn	Lyn	0.804088592529297	3.17309006678365	P25911
basigin	Bsg	0.797182083129883	4.15999122983086	P18572; K3W4Q8; J3QP71
lysosome-associated membrane glycoprotein 1	Lamp1	0.765460968017578	3.04320833845537	P11438
monocyte differentiation antigen CD14	Cd14	0.729209899902344	2.889622737452	P10810
prosaposin	Psap	0.727870941162109	2.15360199268459	E9PZ00; Q8BFQ1; K3W4L3; J3QPG5; Q61207
sodium/potassium-transporting ATPase subunit α-1	Atp1a1	0.721967697143555	4.49493484904153	Q8VDN2
receptor-type tyrosine-protein phosphatase α	Ptpra	0.715917587280273	1.32145282193725	Q91V35; P18052
carbonic anhydrase 2	Ca2	0.700565338134766	2.18240873407234	P00920
cation-dependent mannose-6-phosphate receptor	M6pr	0.694555282592773	1.86549789454771	P24668
guanine nucleotide-binding protein G(*i*) subunit α-2	Gnai2	0.690958023071289	4.49982886978393	P08752; A0A0A6YWA9
solute carrier organic anion transporter family member 4A1	Slco4a1	0.68499755859375	2.62287594512409	Q8K078
60S acidic ribosomal protein P2	Rplp2	0.683738708496094	2.50202473162693	P99027; A0A5F8MPY2
galectin-1	Lgals1	0.678895950317383	2.93679707725021	P16045
Ras-related protein Rab-7a	Rab7a	0.677793502807617	2.27608066338504	P51150; A0A0N4SVG9
protein-tyrosine-phosphatase	Ptprc	0.66557502746582	4.1976947458689	S4R1M0; P06800
macrophage scavenger receptor types I and II	Msr1	0.662124633789063	2.22280175971571	A0A1B0GRS5; P30204
annexin A3; annexin	Anxa3	0.643321990966797	3.93917765945984	O35639; Q3TET3
dolichyl-diphosphooligosaccharide--protein glycosyltransferase subunit 1	Rpn1	0.631629943847656	1.60140194675991	Q91YQ5
integrin β; integrin β-2	Itgb2	0.624547958374023	2.97514378801044	Q542I8; P11835; M0QWA7
guanine nucleotide-binding protein G(*k*) subunit α	Gnai3	0.622499465942383	3.11714466623686	Q9DC51
retinoic acid early inducible protein 1-β	Raet1b	0.622049331665039	3.68057249310752	O08603
brain acid soluble protein 1	Basp1	0.615779876708984	1.59457032964449	Q91XV3
V-type proton ATPase subunit a	Tcirg1	0.614723205566406	2.10278063373262	Q9JHF5
protein EVI2B	Evi2b	0.600425720214844	1.77680526978937	Q8VD58
neuroplastin	Nptn	0.593925476074219	1.7018422710372	H3BIX4; Z4YLB7; P97300; A0A0A0MQ N8; H3BKA7
platelet glycoprotein 4	Cd36	0.591106414794922	3.09538924500363	Q08857; A0A0G2JFB7
lipid phosphate phosphohydrolase 1	Ppap2a	0.590782165527344	1.70627236425915	Q61469
annexin A7	Anxa7	0.588558197021484	1.76746156517526	Q07076; A0A2C9F2D2
V-type proton ATPase subunit C 1	Atp6v1c1	0.587795257568359	1.74946357325171	Q9Z1G3

### Pathway Analysis for Mouse Proteins Enriched in EVs Released by Uninfected or *M*.*ab*-Infected RAW 264.7 Cells

To investigate the potential cellular processes or pathways that are regulated by *M*.*ab*-infected cell-released EVs in recipient cells, we first performed pathway analysis using the differentially enriched host proteins (Tables 1 and 2) in EVs isolated from *M*.*ab*-infected RAW 264.7 cells vs uninfected cells. To gain a comprehensive understanding of the biological significance of our data set, we performed pathway analysis using three complementary methods: Gene Ontology (GO) Biological Processes, GO Cellular Components, and KEGG Pathways. Each method offers unique insights that, when integrated, provide a multidimensional view of EV-carried proteins in recipient cells. Figure 3A shows the top 20 host biological processes that were highly upregulated in EVs isolated from *M*.*ab*-infected RAW 264.7 cells compared to EVs from uninfected RAW 264.7 cells based on the GO Biological Processes category.^29^ These enriched biological processes are mainly involved in molecule transport, intracellular ion homeostasis, and cellular response to extracellular stimulation, such as the import into cell pathway, the regulation of secretion by cell pathway, the carboxylic acid transport pathway, the regulation of superoxide metabolic process and intracellular monatomic cation homeostasis. Similarly, based on the GO Biological Processes category (Figure 4A), we also found a list of downregulated biological processes in EVs isolated from *M*.*ab*-infected RAW 264.7 cells compared to EVs from uninfected RAW 264.7 cells using those host proteins that are diminished in EVs isolated from *M*.*ab*-infected RAW 264.7 cells compared to EVs from uninfected RAW 264.7 cells (Table S1). Differently, the top 5 downregulated biological processes in EVs from *M*.*ab*-infected cells are the biological processes involved in RNA splicing, chromosome condensation, amino acid metabolic process, aerobic respiration, and dicarboxylic acid metabolic process. As shown in Figure 4A, a number of downregulated biological processes regulate protein translation and metabolism in host cells. Since EVs are released by cells, these enriched GO biological processes reflect the cellular response to *M*.*ab* infection in RAW 264.7 cells in cell culture.

**Figure 3:**
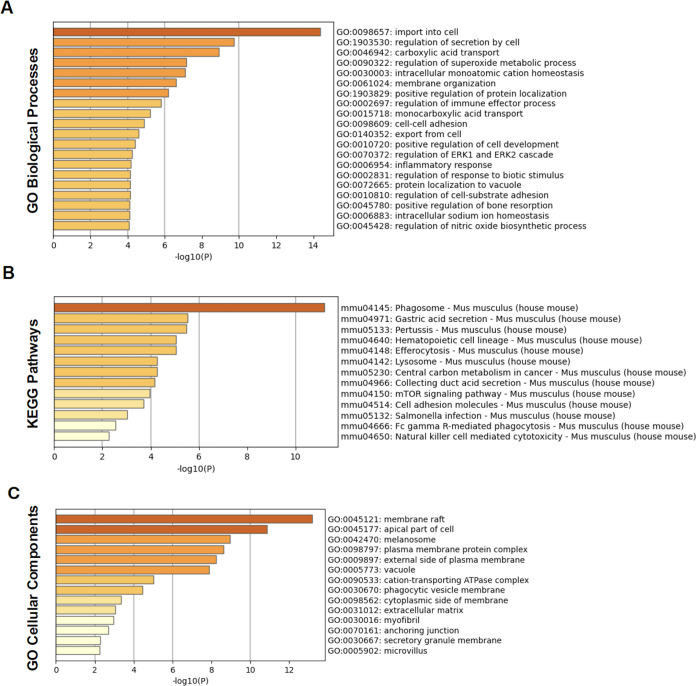
Metascape pathway analysis for host proteins that were upregulated (Tables 1 and 2) in EVs isolated from *M*.*ab*-infected RAW 264.7 cells compared to those from uninfected RAW 264.7 cells. The analysis was performed using various pathway databases. (A) GO biological processes; (B) KEGG; (C) GO cellular components.

**Figure 4:**
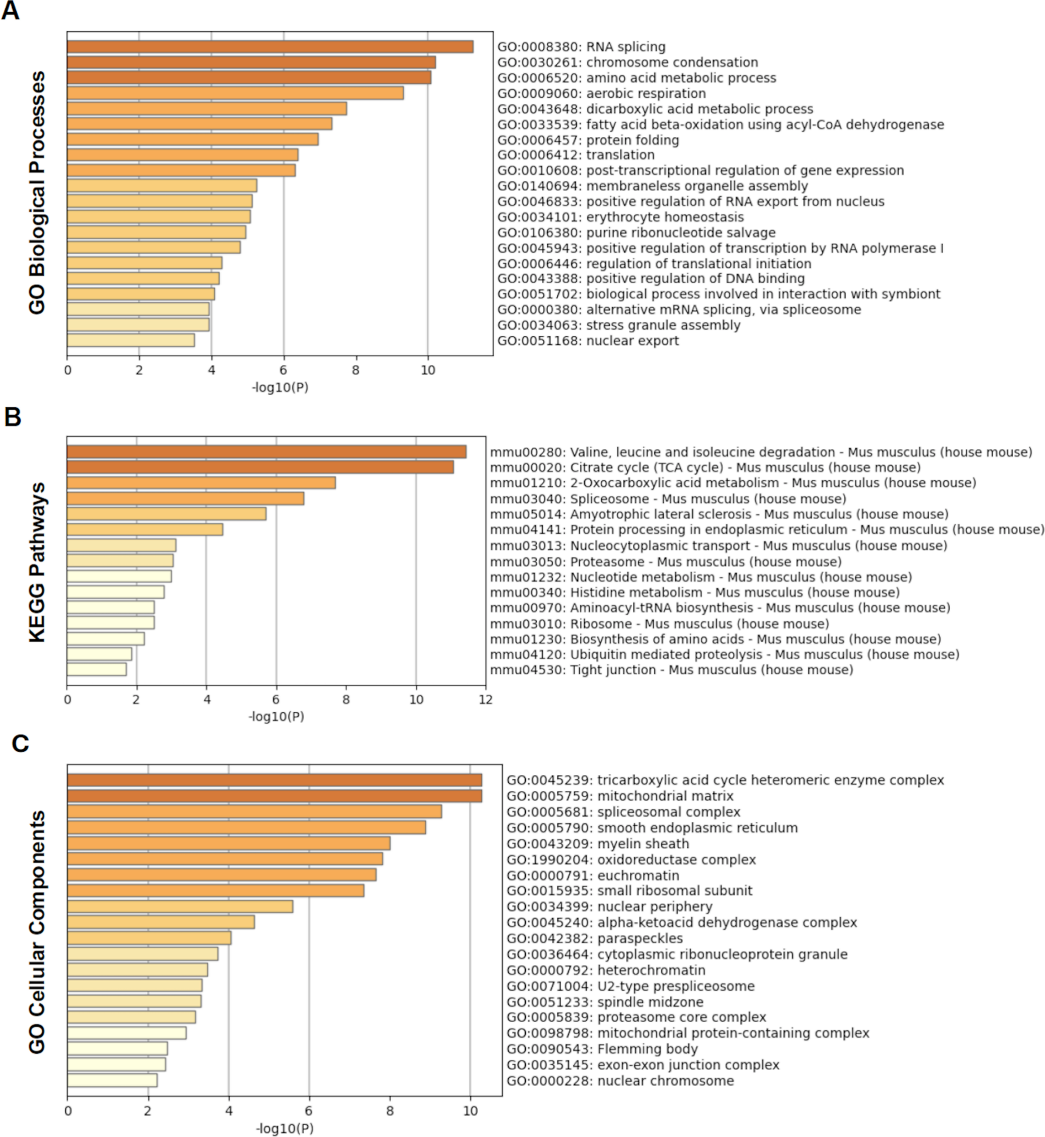
Metascape pathway analysis for host proteins that were downregulated (Table S1) in EVs isolated from *M*.*ab*-infected RAW 264.7 cells compared to those from uninfected RAW 264.7 cells. The analysis was performed using various pathway databases. (A) GO biological processes; (B) KEGG; (C) GO cellular components.

We also conducted pathway analysis based on the KEGG pathway collection (Figure 3B,4B), which mainly determines the interaction between different biological pathways.^30^ Interestingly, we identified several bacterial infection-associated pathways^7,31^ that were highly upregulated in EVs isolated from *M*.*ab*-infected RAW 264.7 cells compared to EVs from uninfected RAW 264.7 cells, including the pathways regulating phagosome and lysosome function, efferocytosis, mTOR signaling, and cellular response to *Salmonella* infection (Figure 3B). Similar to the findings with the GO Biological Processes category (Figure 4A), a number of the downregulated KEGG pathways in EVs isolated from *M*.*ab*-infected RAW 264.7 cells in the KEGG analysis are the pathways engaged in the cellular metabolism and protein translation, such as valine, leucine and isoleucine degradation, citrate cycle (TCA cycle), 2-oxocarboxylic acid metabolism, spliceosome, and protein processing in endoplasmic reticulum (Figure 4B). Like the analysis above in GO Biological Processes, these enriched KEGG pathways reflect the cellular response to *M*.*ab* infection in parental macrophages. Further pathway analysis with the GO Cellular Components Collection, which determines the subcellular localization of proteins,^29^ shows that several host cell plasma membrane-associated pathways were upregulated in EV isolated from *M*.*ab*-infected RAW 264.7 cells compared to EVs from uninfected RAW 264.7 cells, including those involved in the membrane raft, apical part of cell, plasma membrane protein complex, and the external side of the plasma membrane (Figure 3C). Similar to the downregulated pathways enriched using the GO Biological Processes and KEGG collections, a group of downregulated pathways involved in host protein translation and cellular metabolism were identified in EVs isolated from *M*.*ab*-infected RAW 264.7 cells compared to EVs from uninfected RAW 264.7 cells, such as the tricarboxylic acid cycle heteromeric enzyme complex, spliceosomal complex, smooth endoplasmic reticulum, and ribosomal subunits (Figure 4C). It suggests that host proteins involved in protein translation and cellular metabolism were less trafficked into EVs in *M*.*ab*-infected RAW 264.7 cells relative to uninfected RAW 264.7 cells.

In the contrast, the enrichment of the host proteins involved in cytoplastic membrane indicates that *M*.*ab* infection increased the abundance of these proteins in cytoplastic membrane or trafficking to EVs in RAW 264.7 cells.

Metascape network analysis of enriched gene ontology clusters with biological processes reveals that the top 20 upregulated gene ontology clusters in EVs isolated from *M*.*ab*-infected RAW 264.7 cells exhibit high inter- and intracluster similarity (Figure 5A). A similar pattern was observed for the 20 downregulated pathways in EVs isolated from *M*.*ab*-infected RAW 264.7 cells compared to EVs from uninfected cells (Figure 5B).

**Figure 5:**
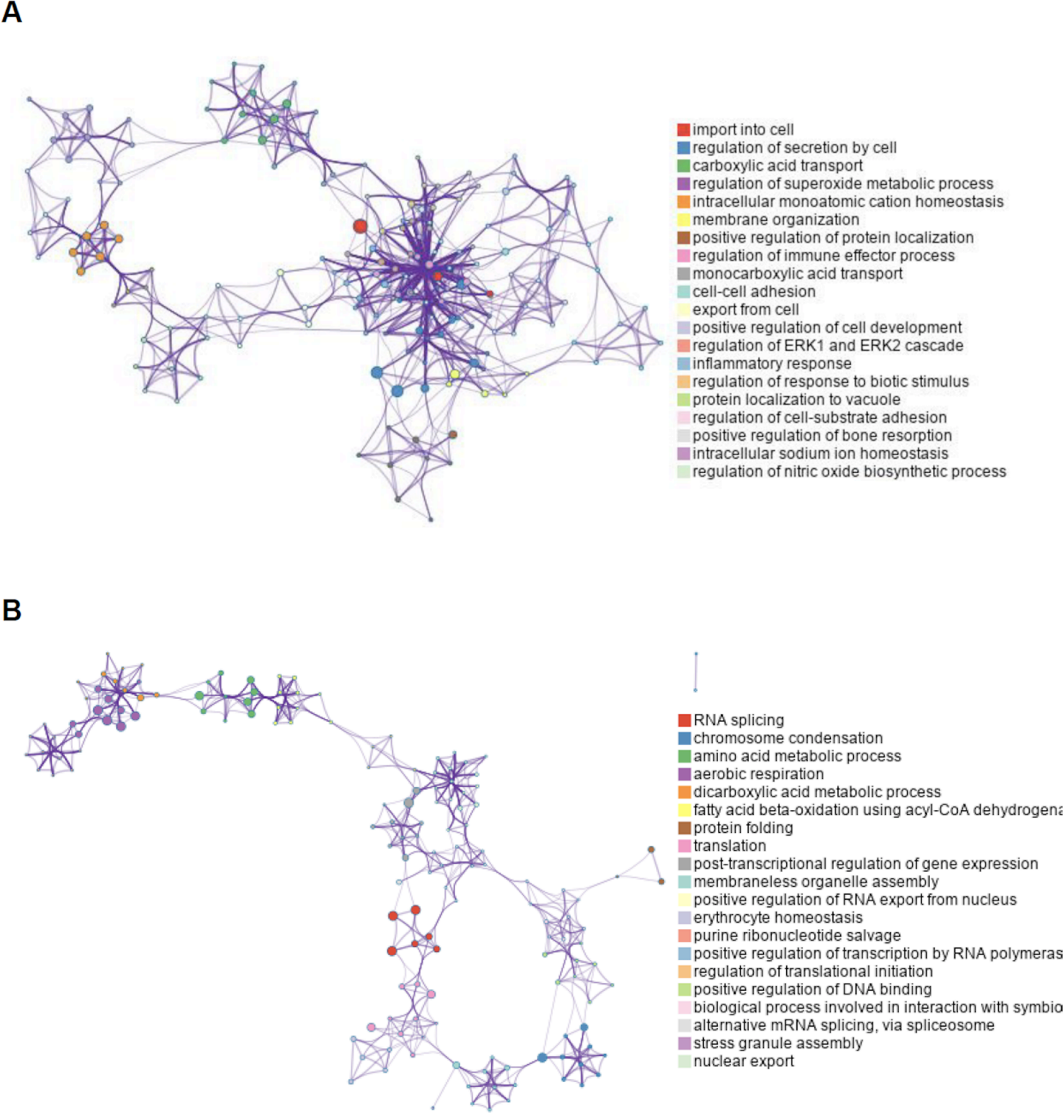
Metascape network for enriched gene ontology clusters based on biological processes. The analysis was performed using host proteins that were differentially enriched in EVs isolated from *M*.*ab*-infected RAW 264.7 cells compared to those from uninfected RAW 264.7 cells. (A) Metascape network for enriched ontology clusters (Go: Biological Process) based on the upregulated mouse proteins in EVs isolated from *M*.*ab*-infected RAW 264.7 cells. (B) Similar to (A), but using the downregulated mouse proteins in EVs isolated from *M*.*ab*-infected RAW 264.7 cells. Each term is indicated by a circular node. The number of input proteins falling into that term is represented by the circle size, and the cluster identities are represented by colors.

### EVs from *M*.*ab*-Infected RAW 264.7 Cells Improve *M*.*ab* Intracellular Growth in Recipient Macrophages in Cell Culture

To determine the effect of macrophage-released EVs on cellular response to mycobacterial infection in recipient macrophages, we analyzed the *M*.*ab* intracellular survival in RAW 264.7 cells that were pretreated with EVs isolated from the uninfected or *M*.*ab*-infected RAW 264.7 cells. Different from EVs isolated from *M*.*ab*-infected human bronchial epithelial cell line 16HBE14o-,^23^ EVs isolated from *M*.*ab*-infected RAW 264.7 cells significantly improved *M*.*ab* intracellular growth in recipient macrophages at 72 h post infection when compared to the untreated group or EVs from uninfected RAW 264.7 cells (Figure 6). EVs isolated from uninfected RAW 264.7 cells slightly enhanced intracellular growth of *M*.*ab* in macrophages compared to the untreated group; however, the difference was not statistically significant. EVs from either uninfected or *M*.*ab*-infected RAW 264.7 cells did not interfere with *M*.*ab* load at 1 h post infection, suggesting they do not affect the uptake of *M*.*ab* by recipient macrophages in cell culture (Figure 6).

**Figure 6:**
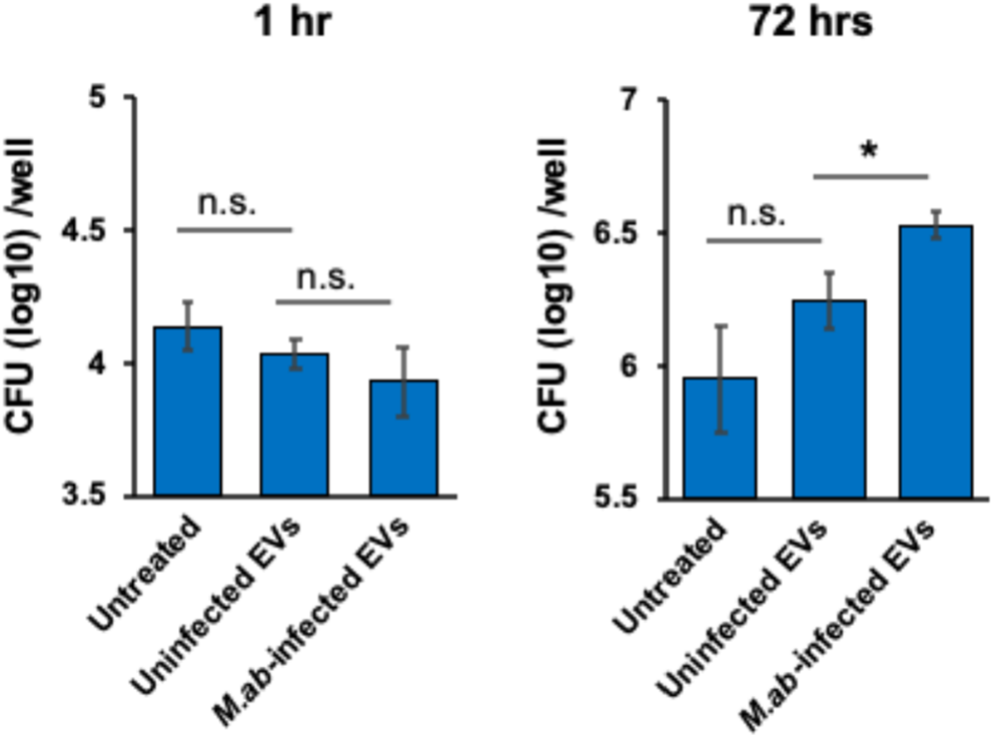
*M*.*ab* survival in mouse macrophages in cell culture. RAW 264.7 cells were pretreated with EVs isolated from uninfected or *M*.*ab*-infected RAW 264.7 cells for 24 h before *M*.*ab* infection (MOI = 5). The results are mean ± SD (*n* = 3/group) and representative of three independent experiments. n.s., not significant; *p < 0.05 by one-way ANOVA, followed by Tukey’s multiple comparisons test.

### EVs from *M*.*ab*-Infected RAW 264.7 Cells Mediate Iron Uptake in Recipient Macrophages

As described above, the gene ontology pathway analysis illustrates that the “Import Into Cell” pathway is the top 1 upregulated host pathway in EVs isolated from *M*.*ab*-infected RAW 264.7 cells relative to EVs from uninfected macrophages (Figure 3A). A heatmap for the proteins engaged in this pathway is shown in Figure 7A. Several amino acid transporter proteins were enriched in EVs isolated from *M*.*ab*-infected RAW 264.7 cells, including Slc3a2, Slc7a5, Slc1a5, Slc2a1, and Slc38a2 (Figure 7A). Interestingly, this enriched pathway also includes the transferrin receptor that is important for intracellular iron uptake in macrophages^32^ (Figure 7A and Table 2). Western blot analysis further confirmed an increased level of transferrin receptor in EVs from *M*.*ab*-infected RAW 264.7 cells compared to EVs from uninfected cells (Figure 7B,7C). Intracellular iron is one of the key nutrients for mycobacterial intracellular growth in macrophages.^19^ It has been reported that the EV membrane can fuse with the cytoplasmic membrane of recipient cells.^15^ Therefore, we hypothesized that EV-carried transferrin receptors likely transfer the transferrin receptors to recipient macrophages and increase iron uptake in these cells.

**Figure 7:**
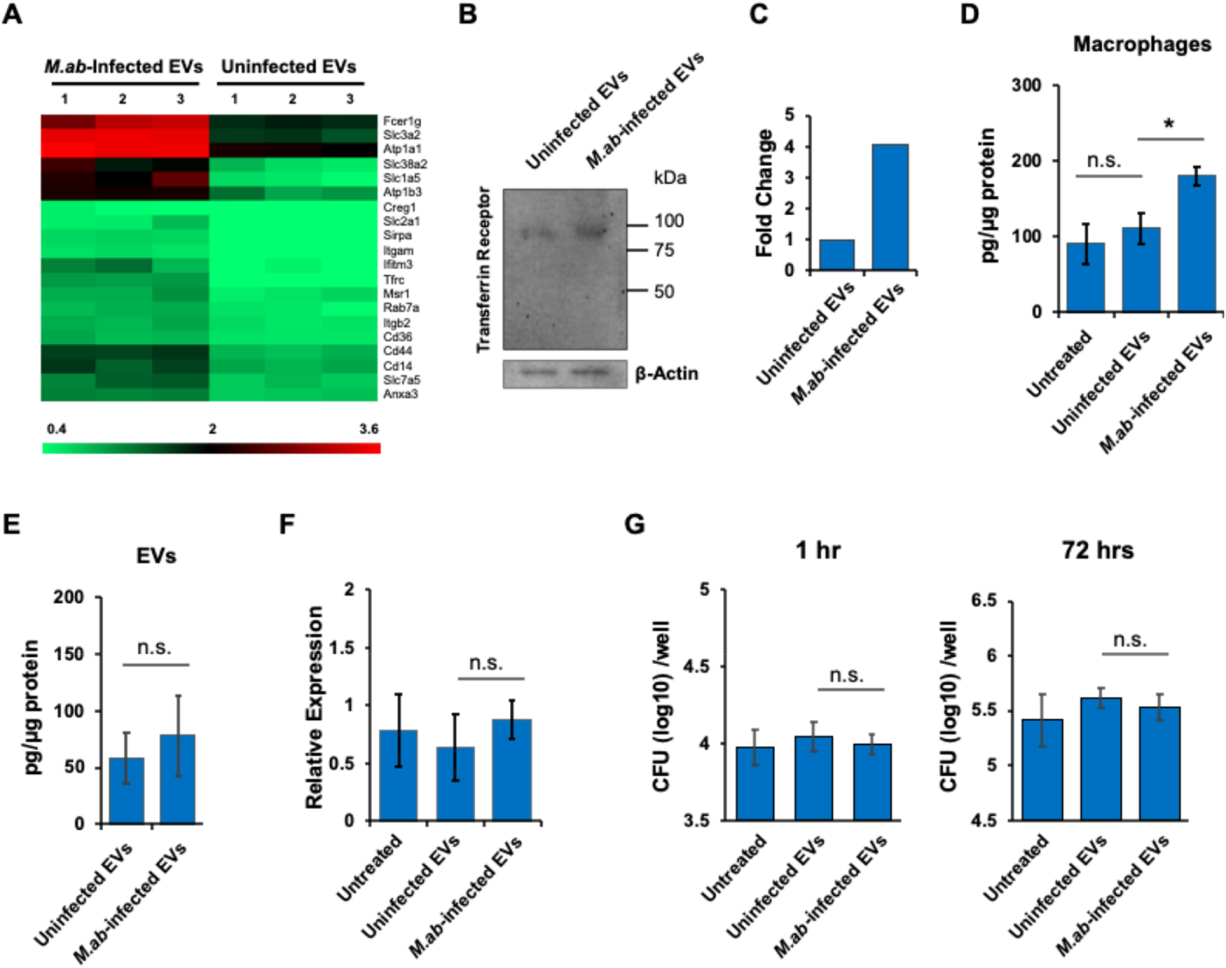
Effect of macrophage-derived EVs on antimycobacterial response in macrophages in cell culture. (A) The heatmap for the host proteins involved in the pathway “import into cell” that were enriched in EVs isolated from *M*.*ab*-infected RAW 264.7 cells vs EVs from uninfected RAW 264.7 cells. (B) Western blot analysis for transferrin receptor (Tfrc) in EVs. β-actin was used as a loading control. (C) Relative abundance of transferrin receptor in EVs based on Western blot analysis. Intensity of target bands was measured by ImageJ and then the intensity of transferrin receptor was normalized to β-actin. The result was expressed relative to uninfected EVs. (D) Intracellular iron abundance in RAW 264.7 cells that were treated with EVs isolated from uninfected or *M*.*ab*-infected RAW 264.7 cells for 24 h. (E) Iron abundance in EVs isolated from uninfected or *M*.*ab*-infected RAW 264.7 cells. (F) Quantitative RT-PCR for the mRNA abundance of the *Tfrc* gene in RAW 264.7 cells that were untreated or treated with EVs isolated from uninfected or *M*.*ab*-infected RAW 264.7 cells for 24 h. (G) *M*.*ab* CFU assay in RAW 264.7 cells that were pretreated with EVs in the presence of iron chelator, deferoxamine. The results in (D–G) are mean ± SD (*n* = 3 biological repeats/group) and are representative of two independent experiments. n.s., not significant; *p < 0.05 by one-way ANOVA, followed by Tukey’s multiple comparisons test.

To test our hypothesis, we measured the intracellular iron abundance in RAW 264.7 cells untreated or treated with EVs isolated from uninfected or *M*.*ab*-infected RAW 264.7 cells in cell culture. As seen in Figure 7D, EVs isolated from *M*.*ab*-infected RAW 264.7 cells significantly increased intracellular iron abundance in recipient macrophages when compared to the untreated group or EVs from uninfected macrophages. Similar to our observation in *M*.*ab* intracellular growth in macrophages (Figure 6), EVs from uninfected RAW 264.7 cells had no significant effect on intracellular iron abundance in recipient macrophages in cell culture (Figure 7D). Different from EV-treated macrophages, a comparable level of iron load was detected in purified EVs from either uninfected or *M*.*ab*-infected RAW 264.7 cells (Figure 7E). Quantitative RT-PCR analysis indicates that EVs from either uninfected or *M*.*ab*-infected RAW 264.7 cells had no effect on the expression of the transferrin receptor gene, *Tfrc*, in recipient macrophages in cell culture (Figure 7F). These results suggest that the increase in iron levels in recipient macrophages by EVs from *M*.*ab*-infected RAW 264.7 cells is likely due to the transfer of transferrin receptor from EVs to the recipient cells, not due to the upregulation of *Tfrc* gene in the recipient macrophages. To determine if EV-induced iron uptake contributes to an increased *M*.*ab* growth in recipient macrophages, we measured *M*.*ab* growth in RAW 264.7 cells that were pretreated with EVs in the presence of iron chelator, deferoxamine, in cell culture. As seen in Figure 7G, EVs isolated from *M*.*ab*-infected RAW 264.7 cells failed to enhance *M*.*ab* growth in recipient macrophages in the presence of deferoxamine.

## Discussion

Intracellular iron homeostasis within host cells is essential for the survival and proliferation of intracellular bacterial pathogens, such as *M*.*tb* and NTMs, in macrophages.^19,20^ Since iron is a critical nutrient for bacterial pathogens, many bacteria, including *M*.*tb* and NTMs, have evolved survival strategies to hijack iron from host cells to support their growth and maintain cellular processes. These pathogens often acquire iron from host cells by inducing the release of iron from host stores or by directly exploiting the host’s iron supply to fuel their intracellular replication. For example, *M*.*tb* and *M*.*ab* produce and secrete siderophores, carboxymycobactin, that can acquire iron from the iron-binding host proteins.^20,33,34^ Previous studies also showed that transferrin-delivered iron accumulated in mycobacteria-containing phagosomes in mouse macrophages that were infected with pathogenic Mycobacteria, including *M*.*avium* 101 or *M*.*tb* H37Rv, in cell culture.^35^ While this iron accumulation provides mycobacterial pathogens a direct access to iron supply, it could be detrimental to bacterial survival within host cells via iron-dependent reactive oxygen species (ROS) production.^36^ However, mycobacterial pathogens, such as *M*.*tb* and *M*.*ab*, has evolved a mechanism to resist iron-dependent ROS production within macrophages.^37^ In this study, we discovered that *M*.*ab*-infected mouse macrophages release EVs that facilitate iron uptake in recipient macrophages in cell culture. This process enhances the intracellular iron availability in the recipient cells, creating a favorable environment that supports the growth and survival of *M*.*ab* within macrophages (Figure 7). Host cell-derived EVs may represent an important mechanism by which *M*.*ab* manipulates host cell metabolism to promote its own intracellular proliferation within macrophages. Our findings suggest that host cell-derived EVs could serve as a novel mechanism by which *M*.*ab* and potentially other intracellular bacterial pathogens acquire essential nutrients, thereby enhancing their ability to survive and replicate in hostile host environments.

Interestingly, several proteins involved in ferroptosis, a form of programmed cell death driven by iron-dependent lipid peroxidation, are highly enriched in EVs isolated from *M*.*ab*-infected RAW 264.7 cells compared to EVs from uninfected cells. These proteins include Slc3a2, Slc7a5, Slc1a5, Slc38a2, and Slc2a1 (Figure 7A and Table 2). This suggests that *M*.*ab* infection may actively influence the vesicular transport of these critical regulatory proteins, which are involved in maintaining cellular homeostasis and redox balance in recipient cells. Notably, the amino acid transporters SLC1A5 and SLC38A2 play crucial roles in the transport of glutamine across the cell membrane. Once glutamine enters the cell, it is converted into glutamate, which in turn serves as a precursor for glutathione (GSH) biosynthesis. GSH, a major intracellular antioxidant, is involved in maintaining cellular redox homeostasis and plays a critical role in protecting cells from oxidative stress.^38^ In the context of ferroptosis, GSH is essential for the activity of GPX4 (glutathione peroxidase 4), which detoxifies lipid peroxides and thereby prevents ferroptotic cell death. SLC3A2 forms heterodimers with SLC7A11, known as the xCT transporter, which is responsible for facilitating the uptake of cystine in exchange for glutamate. This system is essential for the synthesis of GSH as well, as cysteine is a rate-limiting substrate for GSH biosynthesis. By enabling the efficient import of cystine, the xCT transporter helps to maintain intracellular GSH levels, ensuring cellular protection against oxidative stress and ferroptosis.^38^ In contrast to the protective functions of SLC1A5, SLC38A2, and SLC3A2, the glucose transporter, SLC2A1, appears to have a different role in ferroptosis. Recent studies have indicated that SLC2A1, also known as GLUT1, may promote ferroptosis under certain conditions by enhancing cellular glucose uptake, which can contribute to increased glycolytic flux and intracellular iron accumulation. This shift in metabolism could facilitate the generation of reactive oxygen species (ROS) and lipid peroxides, thus driving the ferroptosis pathway.^39^ Taken together, our proteomic data in this study show that the ferroptosis pathway-associated proteins, that are suppressing or promoting this pathway, are highly enriched in EVs released by *M*.*ab*-infected mouse macrophages compared to EVs from uninfected macrophages. Considering our finding that EVs released by *M*.*ab*-infected macrophages increase intracellular iron abundance in recipient macrophages (Figure 7D), the proteins in EVs released from *M*.*ab*-infected macrophages are likely to have a capacity to balance iron levels for the need of bacterial survival, avoiding ferroptosis. In the future study, it would be warranted to determine if *M*.*ab*-infected macrophage-released EVs induce ferroptosis in recipient macrophages via upregulating iron uptake, and if EV-carried amino acid transporters, such as SLC1A5, SLC38A2, and SLC3A2, overcome iron-induced ferroptosis via enhancing the GSH-GPX4 axis in recipient cells.

In our previous study, we isolated EVs from *M*.*ab*-infected RAW 264.7 cells at 100,000*g* after a differential centrifugation, followed by a CD9, CD63 and CD81affinity-based purification.^21^ Similar to our findings in this study (Figure 7A and Table 2), our previous study demonstrated that the glutamine transporter, SLC1A5 and SLC38A2, were also highly enriched in 100,000*g* EVs released by *M*.*ab*-infected RAW 264.7 cells compared to EVs from uninfected cells. Additionally, these EVs-carried SLC1A5 and SLC38A2 potentially serve as extracellular glutamine eliminator to diminish the availability of glutamine and interfere with glutamine-dependent *M*.*ab* killing in recipient macrophages.^21^ Interestingly, the “Import Into Cell” pathway is upregulated in both 100,000*g* EVs^21^ and 10,000*g* EVs (Figure 7A) released by *M*.*ab*-infected RAW 264.7 cells in cell culture when compared to those from uninfected cells. While 3 common host proteins (SLC1A5, SLC38A2 and SLC2A1) are enriched in both 100,000*g* EVs and 10,000*g* EVs released by *M*.*ab*-infected RAW 264.7 cells, the majority of enriched proteins involved in the “Import Into Cell” pathway are different in 100,000*g* EVs and 10,000*g* EVs. Future studies should more specifically aim to determine the distinct and/or conserved mechanisms by which *M*.*ab*-infected macrophages regulate recipient cell function through different populations of EVs.

In summary, this study demonstrates that *M*.*ab*-infected mouse macrophages release EVs that enhance iron uptake in recipient macrophages, creating a favorable environment for *M*.*ab* intracellular growth in cell culture. These EVs carry a high level of the proteins involved in the ferroptosis pathway, such as SLC3A2, SLC7A5, SLC1A5, SLC38A2, and SLC2A1, which play key roles in amino acid transport, redox balance, and the synthesis of GSH, suggesting that *M*.*ab*-infected macrophage-released EVs may regulate ferroptosis in recipient cells by upregulating the GSH-GPX4 axis. Additionally, the research underscores the need for future studies to explore the distinct mechanisms by which different EV populations regulate recipient cell function, as these vesicles contain enriched proteins that modulate cellular pathways critical for host–pathogen interactions. Finally, it would be important to investigate whether EVs have similar functions in human and mouse macrophages, which would further provide valuable insight to the role of host cell-derived EVs during mycobacterial infection in humans.

## Supplementary Material









## Notes

Heatmap for host proteins that are commonly found in all types of EVs from mammalian cells (Figure S1), and relative to Figure 7B (Figure S2) (PDF)
Host proteins unique or upregulated in EVs from uninfected RAW 264.7 cells vs EVs from *M*.*ab*-infected 264.7 cells (Table S1) (XLSX)
Host proteins that are commonly found in all types of EVs from mammalian cells (Table S2) (XLSX)
Mycobacterial proteins in EVs from *M*.*ab*-infected 264.7 cells (Table S3) (XLSX)

## References

[1] Vermeire C. A., Tan X., Liang Y., Kotey S. K., Rogers J., Hartson S. D., Liu L., Cheng Y. (2024). Mycobacterium abscessus extracellular vesicles increase
mycobacterial resistance to clarithromycin in vitro. Proteomics.

[2] Kotey S. K., Tan X., Kinser A. L., Liu L., Cheng Y. (2024). Host Long Noncoding
RNAs as Key Players in Mycobacteria–Host Interactions. Microorganisms.

[3] Moraski G. C., Cheng Y., Cho S., Cramer J. W., Godfrey A., Masquelin T., Franzblau S. G., Miller M. J., Schorey J. (2016). Imidazo­[1,2-a]­Pyridine-3-Carboxamides
Are Active Antimicrobial Agents against Mycobacterium avium Infection
In Vivo. Antimicrob. Agents Chemother..

[4] Martiniano S. L., Nick J. A., Daley C. L. (2019). Nontuberculous Mycobacterial Infections
in Cystic Fibrosis. Thorac. Surg. Clin..

[5] Griffith D. E., Aksamit T., Brown-Elliott B. A., Catanzaro A., Daley C., Gordin F., Holland S. M., Horsburgh R., Huitt G., Iademarco M. F. (2007). An official ATS/IDSA
statement: diagnosis, treatment, and prevention of nontuberculous
mycobacterial diseases. Am. J. Respir. Crit.
Care Med..

[6] Cheng Y., Schorey J. S., Zhang C. C., Tan X. (2017). Protein Kinase
Inhibitors
as Potential Antimicrobial Drugs Against Tuberculosis, Malaria and
HIV. Curr. Pharm. Des..

[7] Chandra P., Grigsby S. J., Philips J. A. (2022). Immune
evasion and provocation by
Mycobacterium tuberculosis. Nat. Rev. Microbiol..

[8] Johansen M. D., Herrmann J.-L., Kremer L. (2020). Non-tuberculous mycobacteria and
the rise of Mycobacterium abscessus. Nat. Rev.
Microbiol..

[9] Pandey S., Kant S., Khawary M., Tripathi D. (2022). Macrophages
in Microbial
Pathogenesis: Commonalities of Defense Evasion Mechanisms. Infect. Immun..

[10] Floto R. A., Olivier K. N., Saiman L., Daley C. L., Herrmann J. L., Nick J. A., Noone P. G., Bilton D., Corris P., Gibson R. L. (2016). US
Cystic Fibrosis Foundation and European
Cystic Fibrosis Society consensus recommendations for the management
of non-tuberculous mycobacteria in individuals with cystic fibrosis. Thorax.

[11] Daley C. L., Iaccarino J. M., Lange C., Cambau E., Wallace R. J., Andrejak C., Böttger E. C., Brozek J., Griffith D. E., Guglielmetti L. (2020). Treatment of Nontuberculous Mycobacterial Pulmonary
Disease: An Official ATS/ERS/ESCMID/IDSA Clinical Practice Guideline. Clin. Infect. Dis..

[12] Armingol E., Officer A., Harismendy O., Lewis N. E. (2021). Deciphering cell–cell
interactions and communication from gene expression. Nat. Rev. Genet..

[13] Schorey J. S., Cheng Y., McManus W. R. (2021). Bacteria-
and host-derived extracellular
vesicles – two sides of the same coin?. J. Cell Sci..

[14] Welsh J. A., Goberdhan D. C. I., O’Driscoll L., Buzas E. I., Blenkiron C., Bussolati B., Cai H., Di Vizio D., Driedonks T. A. P., Erdbrügger U. (2024). Minimal information
for studies of extracellular vesicles (MISEV2023): From basic to advanced
approaches. J. Extracell. Vesicles.

[15] Sedgwick A. E., D’Souza-Schorey C. (2018). The biology
of extracellular microvesicles. Traffic.

[16] Cheng, Y. ; Schorey, J. S. The Function and Therapeutic Use of Exosomes in Bacterial Infections. In Exosomes; Edelstein, L. ; Smythies, J. ; Quesenberry, P. ; Noble, D. , Eds.; Academic Press, 2020, Chapter 6, pp 123–146.

[17] Cheng Y., Schorey J. S. (2019). Extracellular vesicles
deliver Mycobacterium RNA to
promote host immunity and bacterial killing. EMBO Rep..

[18] Schorey J. S., Cheng Y., Singh P. P., Smith V. L. (2015). Exosomes and other
extracellular vesicles in host-pathogen interactions. EMBO Rep..

[19] Kotey S. K., Tan X., Fleming O., Kasiraju R. R., Dagnell A. L., Van Pelt K. N., Rogers J., Hartson S. D., Thadathil N., Selvarani R. (2024). Intracellular iron accumulation facilitates
mycobacterial infection in old mouse macrophages. Geroscience.

[20] Rodriguez G. M., Sharma N., Biswas A., Sharma N. (2022). The Iron Response
of
Mycobacterium tuberculosis and Its Implications for Tuberculosis Pathogenesis
and Novel Therapeutics. Front. Cell. Infect.
Microbiol..

[21] Vermeire C. A., Tan X., Ramos-Leyva A., Wood A., Kotey S. K., Hartson S. D., Liang Y., Liu L., Cheng Y. (2025). Characterization of
Exosomes Released from Mycobacterium abscessus-Infected Macrophages. Proteomics.

[22] Cheng Y., Schorey J. S. (2013). Exosomes carrying mycobacterial antigens can protect
mice against Mycobacterium tuberculosis infection. Eur. J. Immunol..

[23] Guthrie C. M., Meeker A. C., Self A. E., Ramos-Leyva A., Clark O. L., Kotey S. K., Hartson S. D., Liang Y., Liu L., Tan X., Cheng Y. (2025). Microvesicles
Derived from Human
Bronchial Epithelial Cells Regulate Macrophage Activation During Mycobacterium
abscessus Infection. J. Proteome Res..

[24] Kowal J., Arras G., Colombo M., Jouve M., Morath J. P., Primdal-Bengtson B., Dingli F., Loew D., Tkach M., Théry C. (2016). Proteomic
comparison defines novel markers to characterize
heterogeneous populations of extracellular vesicle subtypes. Proc. Natl. Acad. Sci. U.S.A..

[25] Cheng Y., Kiene N. J., Tatarian A., Eix E. F., Schorey J. S. (2020). Host cytosolic
RNA sensing pathway promotes T Lymphocyte-mediated mycobacterial killing
in macrophages. PLoS Pathog..

[26] Wickham, H. Data Analysis. In ggplot2: Elegant Graphics for Data Analysis; Wickham, H. , Ed.; Springer International Publishing, 2016; pp 189–201.

[27] Zhou Y., Zhou B., Pache L., Chang M., Khodabakhshi A. H., Tanaseichuk O., Benner C., Chanda S. K. (2019). Metascape provides
a biologist-oriented resource for the analysis of systems-level datasets. Nat. Commun..

[28] Guthrie C. M., Tan X., Meeker A. C., Self A. E., Liu L., Cheng Y. (2023). Engineering
a dual vaccine against COVID-19 and tuberculosis. Front. Cell. Infect. Microbiol..

[29] Aleksander S. A., Balhoff J., Carbon S., Cherry J. M., Drabkin H. J., Ebert D., Feuermann M., Gaudet P., Harris N. L., Hill D. P. (2023). The
Gene Ontology knowledgebase in 2023. Genetics.

[30] Kanehisa M., Furumichi M., Sato Y., Matsuura Y., Ishiguro-Watanabe M. (2025). KEGG: biological
systems database as a model of the real world. Nucleic Acids Res..

[31] Pagán A. J., Lee L. J., Edwards-Hicks J., Moens C. B., Tobin D. M., Busch-Nentwich E. M., Pearce E. L., Ramakrishnan L. (2022). mTOR-regulated
mitochondrial metabolism limits mycobacterium-induced cytotoxicity. Cell.

[32] Soares M. P., Hamza I. (2016). Macrophages and Iron Metabolism. Immunity.

[33] Gobin J., Horwitz M. A. (1996). Exochelins of Mycobacterium tuberculosis remove iron
from human iron-binding proteins and donate iron to mycobactins in
the M. tuberculosis cell wall. J. Exp. Med..

[34] Foreman M., Kolodkin-Gal I., Barkan D. (2022). A Pivotal Role for Mycobactin/mbtE
in Growth and Adaptation of Mycobacterium abscessus. Microbiol. Spectr..

[35] Wagner D., Maser J., Lai B., Cai Z., Barry C. E., Zu Bentrup K. H., Russell D. G., Bermudez L. E. (2005). Elemental
analysis of Mycobacterium avium-, Mycobacterium tuberculosis-, and
Mycobacterium smegmatis-containing phagosomes indicates pathogen-induced
microenvironments within the host cell’s endosomal system. J. Immunol..

[36] Slauch J. M. (2011). How does
the oxidative burst of macrophages kill bacteria? Still an open question. Mol. Microbiol..

[37] Ferrell K. C., Johansen M. D., Triccas J. A., Counoupas C. (2022). Virulence
Mechanisms of Mycobacterium abscessus: Current Knowledge and Implications
for Vaccine Design. Front. Microbiol..

[38] Li J., Cao F., Yin H.-l., Huang Z.-j., Lin Z.-t., Mao N., Sun B., Wang G. (2020). Ferroptosis: past, present and future. Cell
Death Dis..

[39] Song X., Liu J., Kuang F., Chen X., Zeh H. J., Kang R., Kroemer G., Xie Y., Tang D. (2021). PDK4 dictates metabolic
resistance to ferroptosis by suppressing pyruvate oxidation and fatty
acid synthesis. Cell Rep..

[40] Perez-Riverol Y., Bai J., Bandla C., García-Seisdedos D., Hewapathirana S., Kamatchinathan S., Kundu D. J., Prakash A., Frericks-Zipper A., Eisenacher M. (2022). The PRIDE database resources in 2022: a hub
for mass spectrometry-based proteomics evidences. Nucleic Acids Res..

