# Supplementary material for: Host Cell-Derived Extracellular Vesicles Regulate Iron Uptake in Recipient Macrophages during Mycobacterium abscessus Infection
Source: J Proteome Res. 2025 Oct 22;24(11):5523–35. doi: 10.1021/acs.jproteome.5c00399 (PMC12604032; doi:10.1021/acs.jproteome.5c00399)
Supplement: Supplementary file 1 [file pr5c00399_si_001.pdf]

Supporting Information for

Host Cell-derived Extracellular Vesicles Regulate Iron Uptake in Recipient Macrophages during *Mycobacterium abscessus* Infection

Aidaly Daniela Ramos-Wolfley[1,2, †], Charlie A. Speelmon[1,2, †], Jing Zhang[1], Carlyn M. Guthrie[1,2], Olivia L. Clark[1,2], Steven D. Hartson[1, 4], Lin Liu[2,3], Xuejuan Tan[1,2], and Yong Cheng[1,2, *]

[1] Department of Biochemistry and Molecular Biology, Oklahoma State University, Stillwater, Oklahoma, 74078.

[2] Oklahoma Center for Respiratory and Infectious Diseases, Oklahoma State University, Stillwater, Oklahoma, 74078.

[3] Department of Physiological Sciences, Oklahoma State University, Stillwater, Oklahoma, 74078.

[4] Center for Genomics and Proteomics, Oklahoma State University, Stillwater, Oklahoma, 74078.

* Corresponding author: ycheng@okstate.edu

Supplementary Materials

1. **Table S1.xlsx:** Host proteins unique or upregulated in EVs from uninfected RAW 264.7 cells *vs* EVs from *M.ab*-infected 264.7 cells.
2. **Table S2.xlsx:** Host proteins that are commonly found in all types of EVs from mammalian cells.
3. **Table S3.xlsx:** Mycobacterial proteins in EVs from *M.ab*-infected 264.7 cells.
4. **Figure S1:** Heatmap for host proteins that are commonly found in all types of EVs from mammalian cells.
5. **Figure S2:** Relative to Fig.7B.

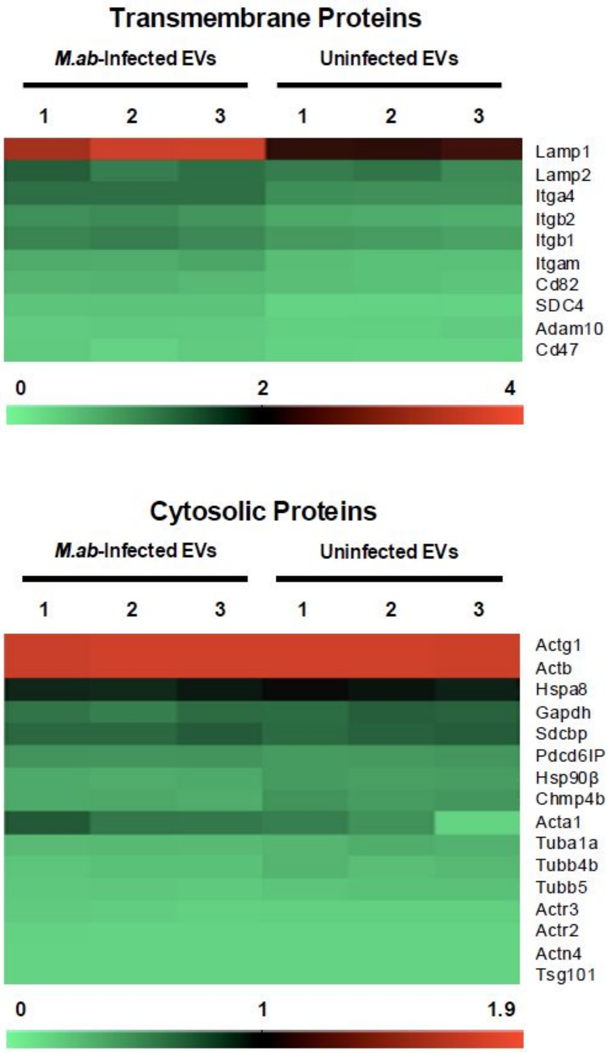

Figure S1: Heatmap for host proteins that are commonly found in all types of EVs from mammalian cells.

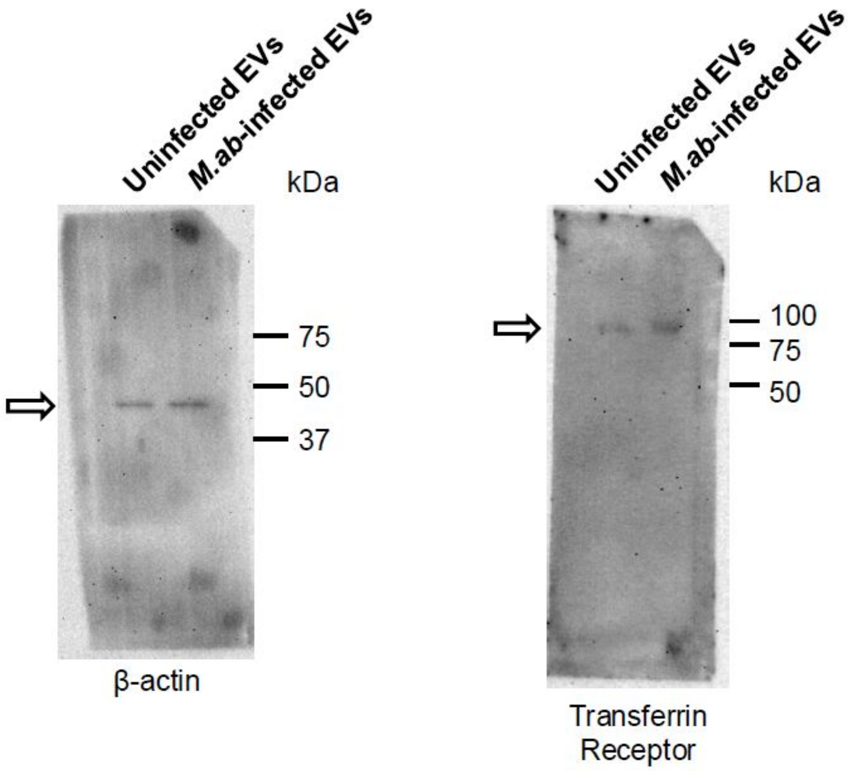

Figure S2: Relative to Fig.7B.
